# Recent Trends in Unpasteurized Fluid Milk Outbreaks, Legalization, and Consumption in the United States

**DOI:** 10.1371/currents.outbreaks.bae5a0fd685616839c9cf857792730d1

**Published:** 2018-09-13

**Authors:** Joanne Whitehead, Bryony Lake

**Affiliations:** Centre for Biomedical Research, University of Victoria, British Columbia, Canada; Meta+ Research and Analysis, British Columbia, Canada

## Abstract

Introduction: Determining the potential risk of foodborne illness has become critical for informing policy decisions, due to the increasing availability and popularity of unpasteurized (raw) milk.

Methods: Trends in foodborne illnesses reported to the Centers for Disease Control in the United States from 2005 to 2016 were analyzed, with comparison to state legal status and to consumption, as estimated by licensing records.

Results: The rate of unpasteurized milk-associated outbreaks has been declining since 2010, despite increasing legal distribution. Controlling for growth in population and consumption, the outbreak rate has effectively decreased by 74% since 2005.

Discussion: Studies of the role of on-farm food safety programs to promote the further reduction of unpasteurized milk outbreaks should be initiated, to investigate the efficacy of such risk management tools.

## Introduction

Current information regarding the risks and benefits of unpasteurized (raw) dairy products is important for decisions regarding food safety policy, and is especially relevant given increasing demand for locally sourced, unprocessed foods, as well as in light of accumulating evidence for health benefits of consuming unpasteurized milk. While nutrient loss on pasteurization of milk is slight 1, cross-sectional studies from several countries have now reproducibly demonstrated significant beneficial immunological effects of unpasteurized milk consumption, primarily protection against childhood asthma, atopy and respiratory illnesses 2^, ^3^, ^4^, ^5^, ^6^, ^7^, ^8 . Several of these studies, including the PARSIFAL study of 15,000 children in five countries 9^, ^10^, ^11, and the GABRIEL study of 10,000 children in three countries 12^, ^13^, ^14^, ^15^, ^16 , were designed to identify determinants of the “farm effect” by which children exposed to farming environments have lower incidence of infections and allergic disorders. Further evidence for the preventive effects of raw milk exposure, both *in utero* and during infancy and childhood, was found from prospective birth cohort studies, notably the six country PASTURE birth cohort of over 900 children monitored until age 6. This study confirmed the strong protective effect of early raw milk consumption against asthma and atopic diseases, and provided insight into mechanisms of immunomodulation 17^, ^18^, ^19^, ^20^, ^21^, ^22^, ^23^, ^24^, ^25^, ^26. Recently, the protective effect of childhood raw milk consumption against adult asthma and atopy, and an association with higher adult lung function, has been demonstrated in the prospective Agricultural Lung Health study of over 3,000 farmers and their spouses in the United States 27^, ^28 . Studies on human cell lines and mouse models are being used to determine the relevant milk components and to further elucidate cellular and genetic mechanisms of action 29^, ^30^, ^31^, ^32 .

This new body of evidence suggests that, given the potential for significant public health benefits which could be gained from a reduction in immunological disorders, a re-evaluation of the risk/benefit profile of unpasteurized milk is in order. A growing number of American states have already legalized unpasteurized milk via farm-gate sales, retail sales, herdsharing, or licensed pet food sales, yet legislators must weigh growing consumer demand against the risk of outbreaks of illnesses due to milk-borne pathogens. Two previous studies 33^, ^34 analyzed state-specific outbreak and legalization data from the United States from 1993 to 2006 and from 2007 to 2012, respectively. Comparing reported outbreak rates in states which provide consumers with legal access to unpasteurized fluid milk vs. states without legal access, both studies concluded that further legalization of unpasteurized milk would likely result in increased numbers of outbreaks. As data on reported outbreaks through 2016 is now available from the Centers for Disease Control and Prevention (CDC), this paper continues the analysis.

## Methods

**Outbreak data collection and classification**

A foodborne disease outbreak is defined as an incident in which two or more people experience a similar illness resulting from the ingestion of a common food 35. Outbreaks in the United States are reported by states to the National Outbreak Reporting System (NORS) of the Centers for Disease Control and Prevention (CDC) 36. Applications were submitted to request NORS outbreak data from 2005 to 2016, related to dairy foods and total outbreaks. The start date of 2005 was chosen as reporting might have been less complete during the first years of the eFORS system, which replaced paper forms in 1998. The field “EstimatedPrimary” provided values for number of illnesses associated with each outbreak. The field and value of “IFSACLevel2=Dairy” was used to extract a set of dairy-related outbreaks; note that this excludes outbreaks involving multiple food types (e.g. ground beef + fluid milk).

Dairy-related outbreak records were reviewed and classified as pasteurized or unpasteurized, processed or unprocessed, and whether a dairy species other than cow was involved. Additional information to clarify contaminated ingredients and pasteurization status was obtained as necessary from the CDC 37^, ^38^, ^39, state health departments 40^, ^41^,^ 42^, ^43^, ^44, *Morbidity and Mortality Weekly Report *45 ^, ^46 ^, ^47, and a third-party website 48^, ^49^, ^50^, ^51^, ^52^, ^53^, ^54^, ^55.

Calculations related to outbreaks and legalization in this paper focus specifically on outbreaks involving unpasteurized fluid milk, excluding those related to processed dairy products (e.g. cheese). For purposes of this analysis, “processing” is defined as including methods which result in a change in consistency of the final product, including inoculation, incubation, condensation, and dehydration; but excluding pasteurization, homogenization, or the addition of flavourings. Unpasteurized milk and cheese as consumables are regulated differently under state and federal law. For example, unpasteurized cheese is legal for sale and interstate distribution if aged as specified in federal regulations 56, while laws governing the sale of unpasteurized fluid milk vary by state but interstate distribution is illegal 57 .

**Data quality issues**

NORS data for 2009 was excluded from outbreak trend analyses due to data quality limitations specific to that year. The CDC’s 2012 report *National Outbreak Reporting System: An Evaluation of Foodborne Disease Outbreak Surveillance and Technical Requirements for Reporting* notes that there was a decrease of almost half of the previous five-year average for all outbreaks, which may have been caused by: “1) technical issues associated with the introduction of the new system, 2) reassignment of types of outbreaks previously reported as foodborne to another mode of transmission, 3) staffing and budgetary issues … or 4) other, unidentified reasons” 58.

A second data quality issue involves errors, inconsistencies and omissions in NORS data. In record CDCID 15533 for example, the value for the field “ExposureState” is recorded as “Pennsylvania” whereas the correct value is “Multistate.” Of 27 records related to queso fresco cheese, six (CDCIDs 2275, 3822, 12355, 14760, 259206, and 268763) were recorded under “IFSACLevel3” as being “Fluid milk” and the other 21 as “Solid/semi-solid dairy products.” Other records contained blank fields or were ambiguous, e.g. in record CDCID 15143 the field indicating pasteurization status (IFSACLevel4) was blank and the implicated food is described simply as “milk.” Additional information was sought when necessary, as described above, and all corrections were confirmed with CDC staff and are detailed in Supplementary Table 1.

**State population scaling**

Annual population estimates from 2005 to 2016 were obtained from the U.S. Census Bureau for each of the 50 states plus the District of Columbia^59^^, ^60. DC has been included as a separate jurisdiction in calculations related to outbreaks and legal status for three reasons: it has a law prohibiting raw milk distribution 61; the CDC lists outbreaks for “Washington DC” as a separate “state”; and it has an estimated population (July 1st 2017 = 693,972) comparable to states such as Alaska (739,795), Vermont (623,657), and Wyoming (579,315).

Population-scaled outbreaks were graphed as outbreaks per million persons for each year, with polynomial regression line fitted in Excel.

**Legal availability of unpasteurized milk**

Information about state laws governing unpasteurized milk distribution was compiled from National Association of State Departments of Agriculture (NASDA) unpasteurized milk surveys for 2004, 2008, and 2011 62^, ^63^, ^64, state government websites 65^, ^66^, ^67^, ^68^, ^69^, ^70^,^ 71^, ^72^, ^73^, ^74^, ^75^, ^76^, ^77^, ^78^, ^79, and third-party sources 33^, ^80^, ^81^,^^82^^, ^83^,^ 84^, ^85^, ^86^, ^87^, ^88^, ^89^, ^90^, ^91^, ^92. A start date of 2004 was chosen as this was the date of the earliest comprehensive NASDA survey.

Five distinct conditions of legal status and distribution were identified and coded: Retail (R; legal off-farm retail and/or farm market sales), Farm-gate (F; legal farm-gate sales but no off-farm sales), Herdshare (H; herdshares permitted by law, written policy, or court order, but sales remain illegal), Pet food (P; farms holding a “pet food” or “commercial feed” permit may sell the product), and Illegal (I; both herdshares and sales are illegal). This system allows for a more precise analysis than the two-group (legal vs. illegal) classification system used by Langer et al. 33 and is less complicated than the seven-group system used by Mungai et al. 34.

Herdsharing is differentiated from selling as it involves legal co-ownership of a herd of dairy livestock by a group of consumers, and states such as Alaska and Colorado (category H) have legalized herdshares while maintaining a ban on sales 66^, ^70 . The 2004 NASDA survey lists herdsharing as being illegal in Alaska; however, this is incorrect as it was legalized in 1998 via a regulatory exemption. In addition to allowing sales, jurisdictions classified in this study as R, F, or P may also have some number of active herdshares distributing raw milk, either licensed and regulated by state law (e.g. Washington State) or unregulated and operating in a legal “grey area” (e.g. California).

It should be noted that the legal status of unpasteurized milk in a jurisdiction is not an exact indicator of accessibility. As an example, some private buying clubs distribute raw milk across state lines, including to jurisdictions such as Virginia, Delaware, and the District of Columbia, where sales are illegal 90.

Though not noted in the two fore-mentioned studies, the legal sale of unpasteurized milk as pet food or commercial feed is included as a separate category. This classification is noted because designation as pet food does not necessarily mean that the primary consumers are animals, as suggested by this Indiana Board of Animal Health statement:

Indiana has a pet food license issued through the State Chemist office at Purdue University. Many raw milk farms have increased sales of their pet food in the form of milk, butter, yogurt, and* cream. *92

In 2008 in North Carolina, a proposed rule requiring dye to be added to pet milk resulted in a successful lobbying campaign by advocates to have the state legislature reverse the rule 93^, ^94. In 2016, the Florida State Department of Agriculture had 84 “registered feed distributors” selling unpasteurized fluid milk to the general public 95. This evidence from three states appears to indicate that classifying unpasteurized milk as “illegal” in states issuing licences for “pet milk” sales may not accurately reflect human consumption patterns within those states.

States were classified where possible according to the actual distribution situation in a state. Accordingly, Nevada has been classified as illegal (I) even though sales are technically legal in that state, as licensing requirements enforce a *de facto* prohibition. A county milk commission which includes a physician and a veterinarian must first be established, committee regulations must be approved by the state, and milk can only be distributed within that county. Only one county milk commission exists, and as of May 2018 no farm had received approval 96. Virginia was classified as Herdshare (H) rather than Illegal (I) as the state permits them on a case-by-case basis 86, with 67 listed on one consumer website 90. Indiana was classified as Herdshare although it also has legal provision for pet food (P), as this appears to be the more common distribution route: the same website lists 30 herdshare farms compared to 7 farms licensed to sell unpasteurized milk as pet food 90. Kentucky and Rhode Island both permit unpasteurized goat milk purchase with a doctor’s prescription, but there is no evidence of this being common practice, so both are classified as Illegal for years in which this was the only legal means of access. For simplicity, the year in which a jurisdiction changed status was assigned the new status.

Access to unpasteurized milk is defined here as “legal” for any jurisdiction categorized as retail, farm-gate, herdshare, or pet food (R, F, H or P).

Foodborne outbreaks may involve multiple states. For between-state comparisons in calculations involving outbreak rates and legalization, the specific states involved in multi-state outbreaks were noted as separate “state-specific outbreak incidents.”

**Unpasteurized milk production licenses**

Numbers of active licensees and permit holders were requested from all state agencies which issue licences and permits to farms to allow them to produce and distribute unpasteurized fluid milk. Where data was not available from state governments, secondary sources such as conference proceedings and media reports were used 63^, ^97^, ^98^, ^99^, ^100^, ^101^,^ 102^, ^103^, ^104^, ^105^, ^106^, ^107^,^ 108^, ^109^, ^110^, ^111^, ^112^,^ 113^, ^114^, ^115^, ^116. Missing data were estimated by interpolating from confirmed numbers.

## Results

**Total reported outbreaks**

Over the twelve year period from January 1 2005 to December 31 2016, there were 10,965 reported foodborne disease outbreaks, resulting in 208,734 illnesses, 10,585 hospitalizations, and 233 deaths. Outbreak information for different food categories is displayed in Table 1. Food vehicles were only identified in 5,236 (48%) of all outbreaks (FB_FoodMain: FoodVehicleUndetermined = "FALSE"), with the foods implicated in the remaining 5,729 outbreaks (52%) undetermined. A caveat should therefore be made that this or any other comparative analysis which uses NORS outbreak data should be viewed in light of the fact that implicated foods remain undetermined in a large proportion of reported outbreaks.

Of outbreaks with identified food vehicles, 232 (4.4%) involved dairy foods (Supplementary Table 1), resulting in 9.2% of all illnesses.

Pasteurized dairy products were responsible for 32 dairy-related outbreaks (13% of all dairy), 2,225 illnesses (45%), 120 hospitalizations (26%), and 17 deaths (74%). Of the 232 dairy-related outbreaks, the pasteurization status of 17 could not be determined. Unpasteurized fluid milk was associated with 152 outbreaks (66% of all dairy), resulting in 1,735 illnesses (35%), 169 hospitalizations (38%), and two deaths (9%). Four of the 152 unpasteurized dairy outbreaks involved both fluid milk and cheese products made from the same milk. Five of the 152 outbreaks involved only goat milk and two involved both cow and goat milk.

Table 1 also displays the average number of deaths per thousand illnesses. Unpasteurized fluid milk shows a similar figure (1.2) to total foodborne outbreaks (1.1), pasteurized dairy is higher at 7.6, and dairy products which are both pasteurized and processed are significantly higher, at 40 deaths per thousand illnesses; this issue will be explored in a follow-up study.

**Figure:**
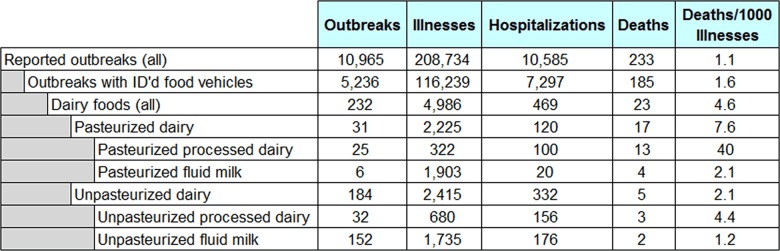
**Table 1:** Reported Foodborne Disease Outbreaks: Total Outbreaks and Dairy Categories from 2005 to 2016. Data from the Foodborne Disease Outbreak Surveillance System, Centers for Disease Control and Prevention.

**Twelve-year review of outbreak trends**

Excluding data for 2009 (5 outbreaks), annual reported outbreaks related to unpasteurized fluid milk started at a low of 10 in both 2005 and 2006 and rose to peak at 18 in both 2010 and 2011. After this peak, outbreaks then saw a general decrease: 14 in 2012, 16 in 2013 and 2014, 11 in 2015, and 13 in 2016. This results in an annual average of 14 outbreaks for the most recent 5 year span, from 2012 to 2016 inclusive.

To analyze outbreak trends, it is necessary to control for changes in population size. When scaled using U.S. Census Bureau population estimates, outbreaks associated with unpasteurized fluid milk increased from 0.034 outbreaks per million persons in 2005 to 0.058 per million in 2010, then decreased to 0.040 per million by 2016. This equates to a 19% percent increase from 2005 to 2016, and a 30% decrease from 2010 to 2016 (Figure 1). The 2009 value (0.016) is less than half of the average of the preceding three and following three years, illustrating the noted data integrity issue for that year.

**Figure:**
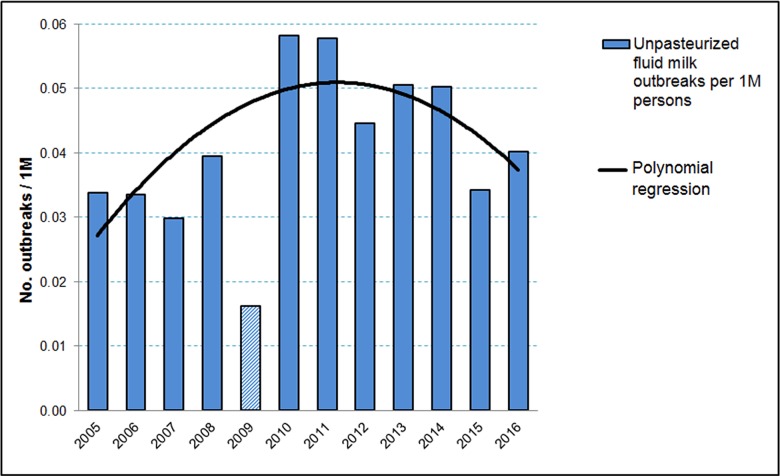
****Figure **1:** Outbreaks associated with unpasteurized fluid milk per million persons; plotted in Excel showing polynomial regression trend line. Outbreaks reported for 2009 provided to illustrate data quality issues specific to that year but excluded from trendline and further calculations. Data from the U.S. Census Bureau population estimates and the Foodborne Disease Outbreak Surveillance System, Centers for Disease Control and Prevention.

**Outbreak rates and legalization of unpasteurized milk**

Table 2 shows the five categories of legal availability of unpasteurized milk and the total proportion of the U.S. population in 2016 living in jurisdictions classified by each code. Complete information for each of 51 jurisdictions (50 states plus the District of Columbia) for 2004 to 2016 is displayed in Supplementary Table 2 using these codes.

**Figure:**
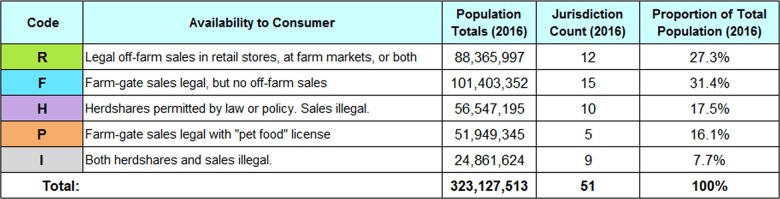
**Table 2:** Legal availability of unpasteurized milk to consumers and percentage of the U.S. population living in states within that category (2016). Data from U.S. Census Bureau population estimates, National Association of State Departments of Agriculture unpasteurized milk surveys, state governments, and third-party websites.

Figure 2 displays a 14 year timeline from 2004 to 2017 of the total number of jurisdictions in each category as defined in Table 2. The total number of jurisdictions providing consumers with legal access to this product (R, F, H & P) increased from 32 in 2004 to 43 in 2017 (+34%); conversely, the number of jurisdictions with no legal distribution (I) fell from 19 to 8 (-58%). Jurisdictions permitting herdshares while banning sales (H) increased from 4 to 11 (+175%), off-farm sales (R) increased from 10 to 12 (+20%), while states permitting farm-gate sales (F) varied between 13 and 15. States where unpasteurized milk is mainly available as pet milk (P) increased in 2016 from 4 to 5 when Maryland began granting permits to farms 81. No state passed laws to restrict or remove accessibility during the study period.

**Figure:**
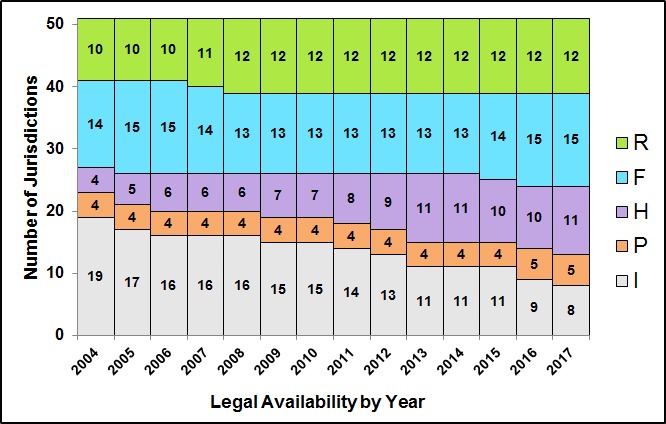
**Figure 2:** National trends in the legalization of unpasteurized milk in the U.S. (2004-2017), applying the legalization categories defined in Table 2. Legal status data from state governments, National Association of State Departments of Agriculture (NASDA) unpasteurized milk surveys, and third-party websites.

In order to analyze the relationship between outbreaks and legalization within and between jurisdictions, the specific states involved in six multi-state outbreaks (CDCIDs 11065, 15533, 257838, 261453, 262220, and 268599) were identified (Supplementary Table 3). As an example, outbreak CDCID 262220 is associated with illnesses in three states: Illinois, Indiana, and Michigan. For purposes of examining outbreak rates vs. legalization within these states, this outbreak was recorded as involving three “state-specific outbreak incidents.” Excluding 2009 data results in the 156 state-specific unpasteurized fluid milk incidents used in further analysis.

Outbreaks per million persons from Figure 1 are plotted in Figure 3 against the number of states which provided consumers with legal access via sales or herdsharing during the twelve year period from 2005 to 2016. With a Pearson’s correlation coefficient of 0.26 (95% confidence interval: -0.40 to 0.74), analysis of the CDC outbreak data for 2005 to 2016 does not support the suggestion that increased legal access to unpasteurized milk leads to higher outbreak rates.

**Figure:**
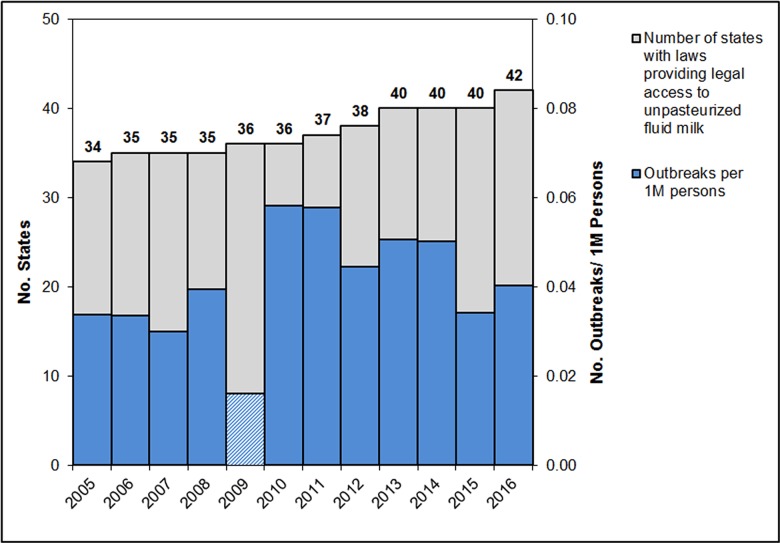
**Figure 3:** States permitting legal access to unpasteurized fluid milk compared to outbreak rates in the United States, 2005-2016. Outbreak data from the Foodborne Disease Outbreak Surveillance System, Centers for Disease Control and Prevention. Population data from the U.S. Census Bureau.

To further examine the hypothesis that legalization leads to higher outbreak rates, one can examine whether a change in legal status within a specific jurisdiction affects the outbreak rate within that jurisdiction. The legal status of unpasteurized milk changed in eleven states during the 12 year period from 2005 to 2016. Six states where raw milk distribution was illegal legalized herdsharing (Kentucky, Michigan, North Dakota, Ohio, Tennessee, and West Virginia), two states with farm-gate sales legalized retail sales (South Carolina and Utah), one illegal state legalized pet food sales (Maryland), one state with legal herdsharing then legalized farm-gate sales (Illinois), and one illegal state first legalized herdsharing and then legalized farm-gate sales three years later (Wyoming).

Five of these states (Kentucky, Michigan, North Dakota, Tennessee, and Wyoming) legalized unpasteurized fluid milk distribution during the middle part of the study period, such that at least four years of outbreak data are available prior to and following legalization (excluding 2009 due to data quality issues), classifying the year in which legalization occurred as “After”. As the subset of data is small, outbreak-incident rates for these five states have been averaged over the four-year before and after periods, shown in Table 3. There was no change in absolute number of outbreaks (6 outbreaks in each 4 year period) and a slight reduction in relative outbreak rates due to population growth during this time.

**Figure:**
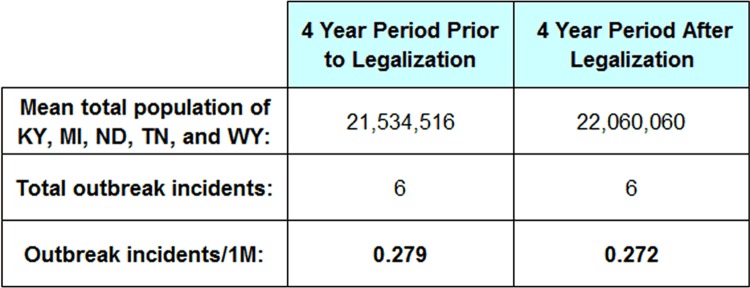
**Table 3:** Outbreak incidence rates per million persons before and after legalization of unpasteurized fluid milk in five states for which there is at least four years of outbreak data prior to and after legalization. Outbreak data from the Foodborne Disease Outbreak Surveillance System, Centers for Disease Control and Prevention. Population data from the U.S. Census Bureau.

Figure 4 shows the average annual outbreak incidence rate per million persons in the U.S. by legal status of unpasteurized milk. Significant variability is seen both over time and between categories of legalization, in part representing fluctuations due to the small absolute number of outbreaks per year within each category. While the Retail category shows the highest overall level of outbreaks, there has been a clear downward trend since 2012. Similarly, the outbreak rate for Farm-gate states peaked in 2011 and has remained low since 2012. The highest variability is seen in the Herdshare and Illegal categories, potentially due to the lack of regulatory oversight in these states.

**Figure:**
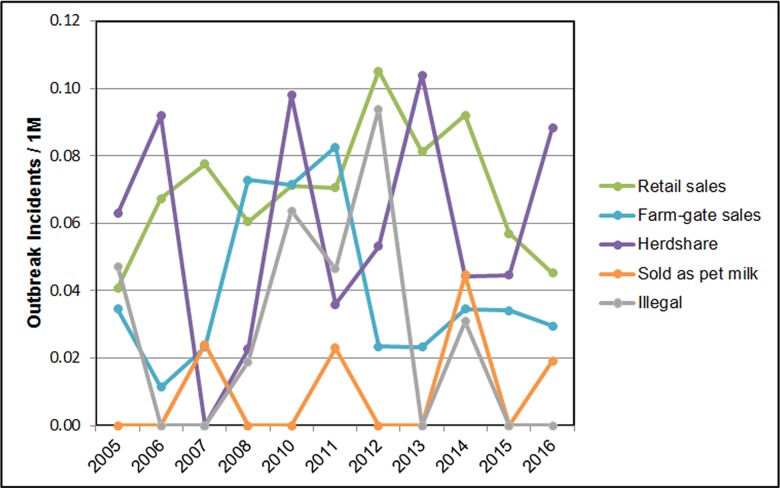
**Figure 4:** Outbreak incidence rate per million persons by legal status of unpasteurized milk, 2005 to 2016 excluding 2009. Outbreak data from the Foodborne Disease Outbreak Surveillance System, Centers for Disease Control and Prevention. Population data from the U.S. Census Bureau.

This summary of outbreak rates by legal status of the state (Figure 4) masks variation between states within each category of legalization. Table 4 illustrates this variation by stratifying states according to average annual outbreak rate over the 12 year period. Within each of the four levels of frequency of outbreaks, there is wide variation on legal availability. Twelve states did not report any outbreaks from 2005 to 2016, six of which provide legal access to unpasteurized milk. While variability is necessarily expected due to the small absolute numbers of outbreaks reported per state, as above, these variations demonstrate that the legal status of unpasteurized milk is clearly not the only determinant of outbreak rate within a state.

Variation also occurs over time within states. As an example, Vermont had the highest average annual outbreak rate at 0.436 outbreak incidents per million persons; however, Vermont had no reported outbreaks from 2011 to 2016. Similarly, Washington State (0.083) had no reported outbreaks from 2012 to 2016.

**Figure:**
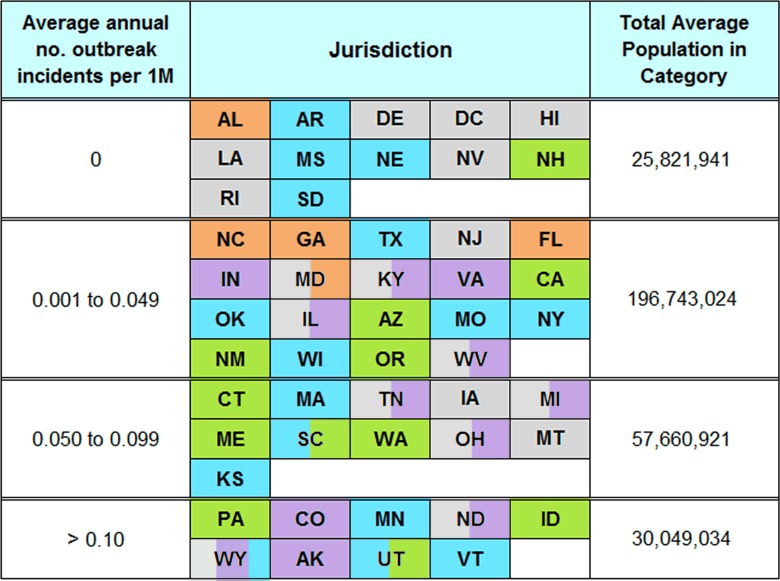
**Table 4: **States stratified by average annual outbreak incident rates for 2005-2016 with 2009 excluded. Outbreak data from the Foodborne Disease Outbreak Surveillance System, Centers for Disease Control and Prevention. Population data from the U.S. Census Bureau.

**Outbreak rates vs. consumption trends**

It is necessary to examine the relationship between outbreak rates and consumption trends in order to determine whether the decline in outbreak rates after 2010 may be due simply to a decline in consumption.

The CDC FoodNet “Atlas of Exposures” 2006/2007 survey indicated that 3.0% of the American population had consumed unpasteurized milk in the previous 7 days 117. However, this survey has not yet been repeated to allow a comparison, nor is there a national reporting system for unpasteurized milk production. It is therefore necessary to use other quantitative measures to estimate trends in consumer demand. One such proxy is the number of state-issued unpasteurized milk farm licences and permits.

Annual totals for the number of licences and permits issued were requested from the fifteen state government agencies which license or issue permits to farms to produce and distribute unpasteurized fluid milk. Adequate data was received from nine of these agencies, representing a diverse geographic area and 40.7% of the total 2016 U.S. population (Table 5). Missing licensing data were estimated by interpolating from confirmed numbers, as indicated by square brackets.

**Figure:**
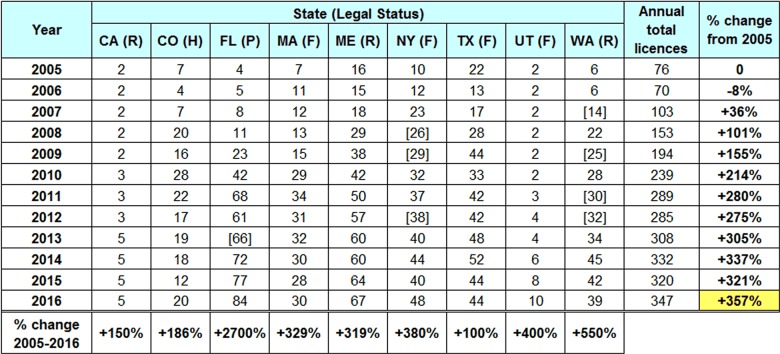
**Table 5:** Number of licences and permits issued to unpasteurized milk farms in nine U.S. states, 2005-2016. Estimates indicated by square brackets. Legal status indicated by colour code as in Table 2. Data from the Foodborne Disease Outbreak Surveillance System, Centers for Disease Control and Prevention, and state government licensing information.

Compiling licence and permit numbers for these nine states shows an increase over twelve years from 76 to 347 licensed unpasteurized milk dairies (+357%). Assuming that licence and permit numbers are a reasonable proxy for consumption, and factoring in U.S. population growth (9.3% over the twelve year period), the ratio between outbreak rate and consumption rate shows a pronounced decline, with the 2016 outbreak-to-consumption ratio only 26% that of 2005 (Figure 5).

**Figure:**
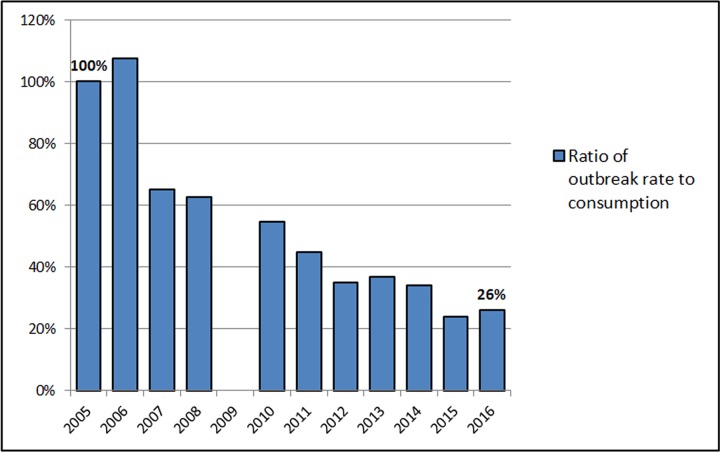
**Figure 5:** Estimated trend in outbreak rate controlling for population growth and estimated consumption rate, 2005-2016, scaled to show values relative to 2005. Data from the Foodborne Disease Outbreak Surveillance System, Centers for Disease Control and Prevention, U.S. Census Bureau population estimates, and state government licensing information.

This calculation represents only an approximate indicator, as only nine states are represented, not all farms producing unpasteurized milk in each state might be licensed, not all licensing agencies maintain an ongoing record of the total number of current active licensees, and changes in farm-specific production levels are not reflected. As an example of how licence numbers may underestimate consumption rates, Missouri reports only one active unpasteurized milk licence 101 while two consumer websites advertise 16 ^118^and 88 current sources of unpasteurized milk within that state, respectively 118^, ^90. As an example of farm-specific change, the Organic Pastures Dairy Company increased production by approximately 60% between 2006 and 2016 (email from Mark McAfee, mark@organicpastures.com, October 5, 2016):

In 2006, we were in 400 stores and 8 farmers’ markets and produced about 680,000 gallons of raw milk per year, versus 2016 with 700 stores, 22 farmers’ markets and 1.09 million gallons .

These examples suggest that our calculation may well underestimate consumption growth, such that the actual decline is likely to be more pronounced than what is shown in Figure 5. The Pearson’s correlation coefficient between number of states with legal access and consumption-scaled outbreak rates of -0.83 (95% confidence interval: -0.96 to -0.47) indicates that, contrary to predictions of previous studies, increased access to and production of unpasteurized milk in the United States over this twelve year period has shown a strong negative correlation with foodborne outbreak rates.

## Discussion

**Comparison with related studies**

By analyzing data from 2007 to 2012, a previous study concluded that “Legalization of the sale of unpasteurized milk in additional states would probably lead to more outbreaks and illnesses” 34. The current study extends that analysis by expanding to cover a twelve year period from 2005 to 2016, controlling for population growth, examining outbreak trends in jurisdictions in which legal status or availability of the product has changed, and estimating changes in consumption during the study period.

In contrast with other studies 33^, ^119, this paper does not include outbreaks caused by unpasteurized cheese. Not only is unpasteurized cheese production and distribution regulated under Federal law and is thus unrelated to state-specific laws regulating unpasteurized fluid milk distribution, but processing itself introduces other factors which could affect relative risk, an issue which will be explored in a separate analysis.

Also in contrast to other studies 34^, ^119, this study excluded 2009 outbreak data from calculations due to data quality issues specific to that year 58. Suggestive of this issue, the figure of nine dairy-related outbreaks in 2009 is less than half of the average annual number (μ=20.3, σ =1.86) for the remaining 11 years in the 12 year period under observation. Using 2009 data in longitudinal analyses of trends could therefore suggest a larger increase relative to that year than what actually may have occurred.

**Outbreaks and Legalization**

Given the four observed trends of a reversal in the number of reported outbreaks (Figures 1 and 3), increasing legalization (Figures 2 and 3), no increase in outbreak rates in five states which legalized raw milk (Table 3), and increasing consumption (Table 5), evidence was not found that supports the position that the legalization of unpasteurized milk within a jurisdiction will cause an increase in outbreaks. Indeed, examining data up to and including 2016 shows that increased legal access after 2010 has been concurrent with generally declining outbreak rates, irrespective of change in consumption. As Figure 4 and Table 4 both illustrate, outbreaks can still occur in states where distribution is illegal, so legal prohibition itself is not a guarantee of consumer safety.

Using the proxy measure of number of licensed unpasteurized milk dairies, consumption was estimated to have increased by 357%. Although this is only suggestive of a general upward trend, a similar increase was recently seen in the United Kingdom, where raw milk consumption in England, Wales, and Northern Ireland increased from 3% in 2012 to 10% in 2018 120.

A decline in outbreaks caused by a particular food vehicle could be due to reduced consumption of that food or to changes in methods of production, processing, and handling. As an example, the 1997 introduction of the HACCP (hazard analysis and critical control points) food safety system in meat processing plants throughout the U.S. was responsible for a 42% decline in *E. coli*-related illnesses over the subsequent seven years 121. Similarly, the decline in unpasteurized milk related outbreaks could be related to changes in product handling or to the implementation of bacterial testing standards and inspection programs. Further studies should explore the efficacy of factors such as education and regulatory programs in preventing outbreaks, and in particular, examine what may have enabled some states (e.g. Vermont and Washington) to reduce outbreak rates to zero while others (e.g. Utah) have seen no decrease (see Supplementary Table 4).

Regarding education, a challenge for dairies has been a lack of on-farm food safety programs. This changed in 2010 when the Farm-to-Consumer Legal Defense Fund made training materials available and the Raw Milk Institute began developing a HACCP-based on-farm food safety program 122^, ^123^, ^124. The decline in frequency of outbreaks coincides with the introduction of these targeted education programs. The tentative conclusion can be drawn that, similar to what was seen in the meat processing industry, the implementation of on-farm food safety systems for unpasteurized milk production may be related to the observed reduction in outbreak rates.

Data from Pennsylvania supports this connection. As shown in Figure 6, outbreaks occurred each year from 2006 to 2014, then no outbreaks were reported for 2015 or 2016, then one outbreak occurred in 2017 125. An interesting correlation is that in November 2014, Pennsylvania State University’s College of Agricultural Sciences hosted a workshop on unpasteurized milk safety in collaboration with the Raw Milk Institute 126^, ^127. In addition, in 2014 the proprietor of the largest Pennsylvania unpasteurized milk farm was trained and listed with the Raw Milk Institute 128. This farm had been responsible for outbreaks in 2012 and 2013 (CDCIDs 15533 and 15482) including one associated with 148 out of 258 (57%) unpasteurized milk related illnesses reported nationwide in 2012 129^, ^130^, ^131. No outbreaks have been associated with this farm’s products since training and listing. While it is impossible to show causation, this correlation of fewer outbreaks with the implementation of a HACCP-based on-farm food safety training program has implications for further studies of the role which education and extension programs may play in the safe production and handling of unpasteurized milk.

**Figure:**
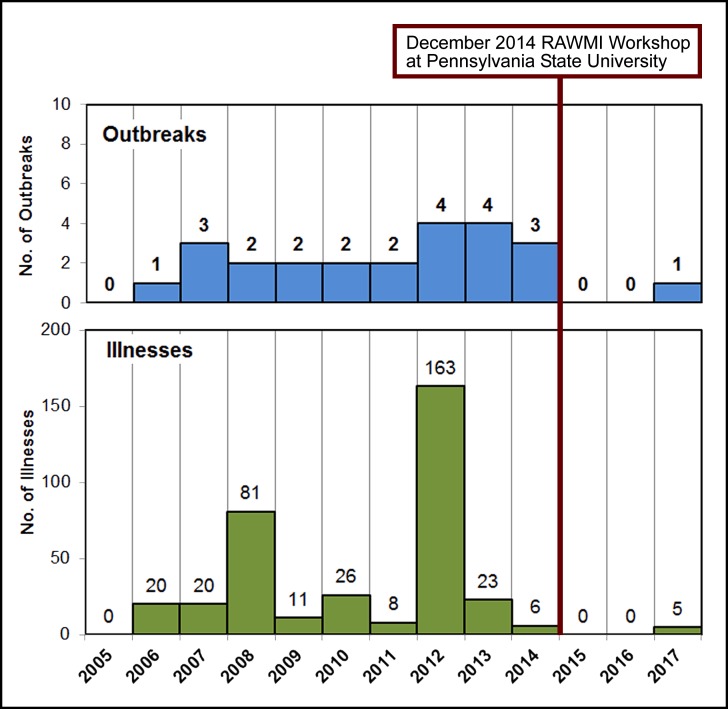
**Figure 6:** Annual number of outbreaks and illnesses related to unpasteurized fluid milk reported in Pennsylvania, 2005-2017. Data from the Foodborne Disease Outbreak Surveillance System, Centers for Disease Control and Prevention and Pennsylvania State Bureau of Epidemiology.

It should be noted that this study does not examine whether or not states which legalized raw milk also implemented mandatory licensing, bacterial testing standards, on-farm food safety plans, or farm inspections by state agriculture or health departments as part of the legalization process. Regulatory programs such as these may well mitigate outbreak rates, and further studies should explore this question. For example, Vermont had three outbreaks between 2008 and 2010, but has had no incidents since. This change coincides with an adjustment period after the introduction in 2009 of a stronger regulatory system 132.

The federal ban on interstate trade 57 in unpasteurized fluid milk has not eliminated either multi-state outbreaks or the emergence of unregulated interstate buying clubs. As 43 states representing 92.6% of the U.S. population currently (March 2018) permit legal access to unpasteurized milk, and legalization does not correlate with increased outbreak rates, expanded legalization along with the development of a federal regulatory framework could be considered, similar to national systems already in effect in other nations such as England, France, Germany, and New Zealand 133^, ^134^, ^135^, ^136.

****Study Limitations****

This study is subject to the limitations of the underlying data, as CDC does not have a record of all outbreaks which have taken place in the United States. Not all outbreaks are identified, investigated, or reported, and the source of foodborne illnesses are often not identified. Even when a single food is identified, the point of contamination is not always known or reported. As documented by the CDC, “The quality of NORS data is dependent on outbreak investigations conducted by state and local health departments, the usability of the reporting system, and the resources available to each state and local health department to investigate and report outbreaks." 58 Nevertheless, the CDC database contains a substantial body of evidence with which to estimate true outbreak rates, and as such, is widely used for public health policy decision-making. In addition, state bacterial standards were not examined in order to determine if these have an effect on outbreak rates, and it is recommended that this be the subject of a future study.

A more significant limitation relates to the difficulty in estimating the actual extent of unpasteurized milk production or consumption. It is apparent from licensing statistics and consumer websites that unpasteurized milk is currently being produced on a larger scale than in past decades; however, with the lack of standardized reporting, and with many farms operating outside of any regulatory structure, any estimate of unpasteurized milk production necessarily incurs a large margin of error. The CDC FoodNet “Atlas of Exposures” survey provided the most comprehensive estimate of unpasteurized milk consumption published to date, but without data beyond 2006/7, there is no record of recent trends.

**Further Studies**

As the variability of state outbreak rates is not simply dependent on legal status, other factors beyond the scope of this study might be affecting the safety of unpasteurized milk production. Topics for further investigation could include: state-specific regulatory structures including inspections and licensing; farm-specific factors such as product handling and pathogen testing; consumer education regarding food safety and the appropriate transport, storage and handling of unpasteurized dairy products; and comparison of milk produced by dedicated raw milk farms with milk from conventional dairy farms doing “incidental sales” of unpasteurized milk to the public. Together, such studies would provide valuable tools to aid in assessing the impact of policy decisions and state or federal unpasteurized milk regulatory structures, thus facilitating evidence-based decision making in public health.

## Conclusions

From a public health perspective, the lack of consistency and comprehensiveness in measuring production or consumption of unpasteurized milk is problematic; however, this analysis provides a current best estimate of the scale of disease outbreaks due to unpasteurized milk. The potential for foodborne illness continues to be a small but real risk from consuming unpasteurized fluid milk, but analysis of data over a twelve year period demonstrates that increased access to this product within the United States has not led to increased outbreak rates. On the contrary, total reported unpasteurized milk-associated outbreaks have declined since 2011, despite increased production, and outbreak rates proportional to estimated consumption rates have declined by 74% over the twelve year period.

The evidence that legalization of unpasteurized milk has correlated with decreased outbreak rates has potential implications for public policy decisions. Recent introduction of on-farm food safety training programs for unpasteurized milk producers may be a factor in the recent decline in outbreak rates. Further studies of the efficacy of such “best-practices” training will be necessary in determining the utility and efficacy of these risk-management options, and could enable the transition from prohibition-based to harm reduction-based regulatory structures. This in turn will enable the further development safe and minimally processed dairy products, to take advantage of the enormous public health benefits that would result from a significantly lower incidence of infections and allergic disorders provided by consumption of fresh, unprocessed milk.

## Data Availability

This study is based on publicly available data from the U. S. Centers for Disease Control and Prevention’s National Outbreak Reporting System (NORS) and the United States Census Bureau. The legal status of unpasteurized milk was determined from National Association of State Departments of Agriculture (NASDA) unpasteurized milk surveys, state governments, and third-party websites. Licensing data were obtained from state governments. Details of all dairy-associated outbreaks, as well as regulatory status and outbreak rate for each jurisdiction and year in the study period are available in the Supplementary Materials.

## Supplementary Information

All supplementary material is available at http://figshare.com/s/866c3d82f50105ff5dab

## Corresponding Author

Joanna Whitehead is the corresponding author and be contact at jowhite@uvic.ca.
